# Acousto-Plasmonic Sensing Assisted by Nonlinear Optical Interactions in Bimetallic Au-Pt Nanoparticles

**DOI:** 10.3390/mi8110321

**Published:** 2017-10-28

**Authors:** Eric Abraham Hurtado-Aviles, Jesús Alejandro Torres, Martín Trejo-Valdez, Guillermo Urriolagoitia-Sosa, Isaela Villalpando, Carlos Torres-Torres

**Affiliations:** 1 Sección de Estudios de Posgrado e Investigación, Escuela Superior de Ingeniería Mecánica y Eléctrica Unidad Zacatenco, Instituto Politécnico Nacional, Ciudad de Mexico 07738, Mexico; ericabrahamh@gmail.com (E.A.H.-A.); guiurri@hotmail.com (G.U.-S.); 2 Academia de Ciencias (Acústica), Escuela de Laudería, Instituto Nacional de Bellas Artes y Literatura, Querétaro 76000, Mexico; jesusalejandrott@yahoo.com.mx; 3 Escuela Superior de Ingeniería Química e Industrias Extractivas, Instituto Politécnico Nacional, Ciudad de Mexico 07738, Mexico; martin.trejo@laposte.net; 4 Centro de Investigación para los Recursos Naturales, Salaices, Chihuahua 33941, Mexico; isaelav@hotmail.com

**Keywords:** two-photon absorption, acousto-optics, CO_2_ detection, surface plasmon resonance

## Abstract

A strong influence of mechanical action in nonlinear optical transmittance experiments with bimetallic nanoparticles integrated by gold and platinum was observed. The nanostructured samples were synthesized by a sol-gel method and contained in an ethanol suspension. UV-VIS spectroscopy evaluations, Transmission electron microscopy studies and input-output laser experiments were characterized. A two-photon absorption effect was induced by nanosecond pulses at 532 nm wavelength with an important contribution from the plasmonic response of the nanomaterials. All-optical identification of acoustical waves was remarkably improved by optical nonlinearities. High sensitivity for instrumentation of mechano-optical signals sensing particular fluids was demonstrated by using a variable carbon dioxide incorporation to the system.

## 1. Introduction

The success of noble metal nanoparticles (NPs) has been empowered by their fascinating improved properties in comparison with bulk materials [1]. The ultrafast optical response dominated by the oscillation of the metal conduction electrons related to the surface plasmon resonance (SPR), has become an exceptional influence on the third-order nonlinear optical (NLO) properties of metallic NPs [2]. The optical properties derived by SPR effects in low-dimensional noble metals (Au, Ag, Pd, Pt, etc.) depend on their morphology, structure, composition and environment [3,4]. Moreover, the size and shape control by means of the preparation methods has direct contribution on physical, chemical, optical, electrical, mechanical, and catalytic processes [5,6]. Optical phenomena exhibited by metallic NPs are related to the excitation characteristics, such as intensity, wavelength, repetition rate, diameter, and pulse duration [7].

Metallic NPs exhibit unique and distinctive chromatic properties which can be analyzed by optical absorption spectroscopy techniques [8]. Among all metallic NPs, gold NPs (Au NPs) have gained much attention for potential bio-nanotechnology and nanomedicine applications. Au NPs are currently employed in different instrumentation fields due to their NLO behavior [9], drug delivery potential [10], and therapeutic effects [11]. Au NPs are excellent materials in terms of photo-thermal conversion mechanisms and internal health monitoring [12]. Concerning the IR exposure of biological systems, as in cancer radiotherapy and photodynamic therapy to fight tumor cells, Au NPs are able to enhance radiation effects via physical, chemical, or biological interactions [13]. The pure Au NPs show a typical absorption band in the visible region, influenced by the SPR associated with the NPs [14]. In contrast, the absorption of platinum NPs (Pt NPs) presents resonances in the ultraviolet region [15], taking advantage of their ability for high-energy photon manipulation to be considered for batteries and fuel cells developments [16]. Pt NPs may give rise to new photo-thermal agents [17] that may be assisted by biostability and adhesion properties that are strongly attractive for nano/micro-engineered systems for neurological diagnostic [18]. Additionally, the electronic properties of Pt NPs make them useful for building optical signal processing devices, such as circuits and electrodes of multilayer ceramic capacitors [19].

In a different way, multimetallic NPs exhibit distinct advantages in comparison with monometallic particles [20,21], since new tailored effects related to the combination of different metal elements can be obtained [22]. Among the bimetallic NPs, Au-Pt NPs have been widely studied due to electrocatalytic properties in special activities for fuel cell reactions, such as methanol oxidation reactions (MOR) and oxygen reduction reactions (ORR) [23]. Additionally, the formation of bimetallic alloy NPs integrated by gold and platinum have recently emerged due to their optical response as selective sensors dictated by the SPR [24]. 

On the other hand, remarkable effects have been identified from the interaction of optical waves with mechanical vibrations [25]. It has been reported that acoustic waves have a significant influence on optical signals with promises in the evolution of instrumentation of quantum phenomena [26,27].

By using a source that provides acoustic vibrations, the propagation and attenuation of sound waves through suspensions could be used to manipulate objects [28]. At nanometric scale, acoustic interactions may produce a predominant vibration response signature of particular NPs [29]. In addition, is effective the participation of third-order optical nonlinearities of nanostructured samples for designing photo-thermal processes [30].

It has been pointed out a large enhancement in the performance of optical systems by recent advances in plasmonics [31,32]. An infinite number of new configurations with different nanomaterials can be considered for nanophotonic and acousto-optical signals processing [33,34]. 

In this direction, this paper has been devoted to study acousto-optic and nonlinear optical properties exhibited by Au-Pt nanoparticles with plasmonic properties associated with their bimetallic nature. Potential applications for instrumentation of mecano-optical signals and gas sensing were experimentally explored. 

## 2. Materials and Methods

### 2.1. Synthesis of Bimetallic NPs

Bimetallic Au-Pt NPs suspended in ethanol were prepared by a previously described sol-gel method [35]. Titanium i-propoxyde [Ti(OC_3_H_7_)_4_] (Sigma-Aldrich, Mexico City, Mexico) was used as a precursor with a concentration C = 0.05 mol/L, pH = 1.25, together with water/alkoxide (Sigma-Aldrich) with a molar ratio rw = 0.8. Au and Pt precursors (Sigma-Aldrich) were used in a similar nominal metal concentration of 1000 mg/L each. We employed a 0.76% (mol/mol) ratio of the (Au + Pt)/Ti(OC_3_H_7_)_4_ mixture suspended in a 11.5 mL volume. The bimetallic Au-Pt NPs were obtained by the assistance of an ultraviolet light reactor.

### 2.2. Acousto-Plasmonic Sensing

**Figure 1:**
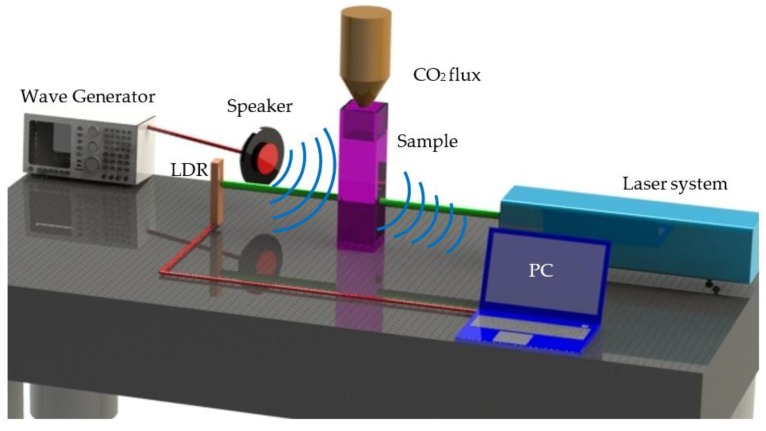
Experimental setup for nonlinear acousto-optical measurements.

Figure 1 illustrates the experimental setup employed to explore acousto-optical effects by single-beam transmittance observations in NLO materials. A Nd:YAG laser system Continuum Model SL II-10 (Continuum, Vancouver, Canada) was used to provide optical waves featuring green light at 532 nm wavelength. A pulse duration of 4 ns with 100 mJ of pulse energy was employed for the experiments. The repetition rate was 1 Hz and the beam diameter was 6 mm.

Acoustic waves were controlled by an electronic generator connected to a speaker. The sample of Au-Pt NPs suspended in ethanol was confined in a quartz cuvette with 1 mm thickness. The optical detection was performed by using a LDR photoresistor (Arduino, Ivrea, Italy).

Alternatively, Figure 1 also illustrates the possibility to incorporate the influence of a CO_2_ flux instead of electronically-generated acoustical waves. A Princeton Applied Research Model 616A Electrode Rotator (AMETEK, Inc. Berwyn, PA, USA) connected to a potentiostat was used for delivering CO_2_ in the experiment. The CO_2_ flux was controlled by a Cole-Parmer correlated flowmeter with values from 0 to 300 PSI. As the same way, optical data acquisition was carried out by a LDR photoresistor.

## 3. Results and Discussion

**Figure 2:**
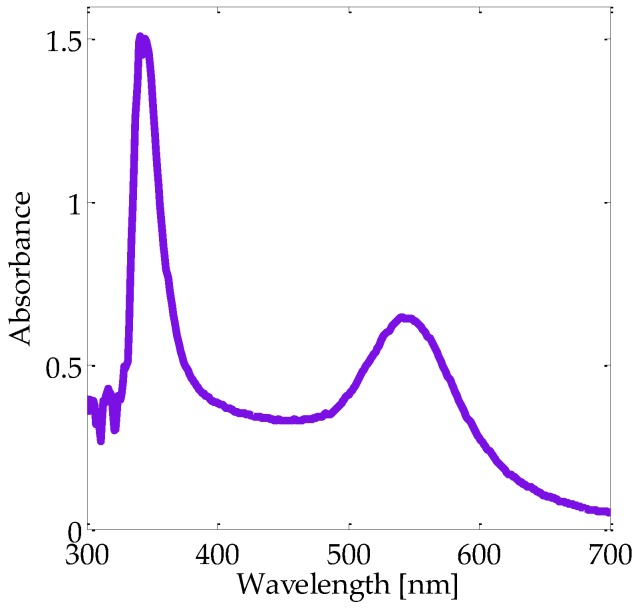
Optical absorption spectrum of Au-Pt NPs suspended in an ethanol solution.

The metallic concentration in the liquid solution was heuristically chosen to clearly observe the absorption bands related to the SPR of the bimetallic Au-Pt NPs by UV-VIS spectroscopy. In Figure 2 can be seen that the two peaks that indicate the formation of an alloy plasmonic structure that correspond to Au and Pt elements. The absorption band close to 550 nm wavelength can be associated with the plasmonic response of the Au NPs while the Pt NPs give rise to the SPR peak near the 350 nm wavelength in the plot.

**Figure 3:**
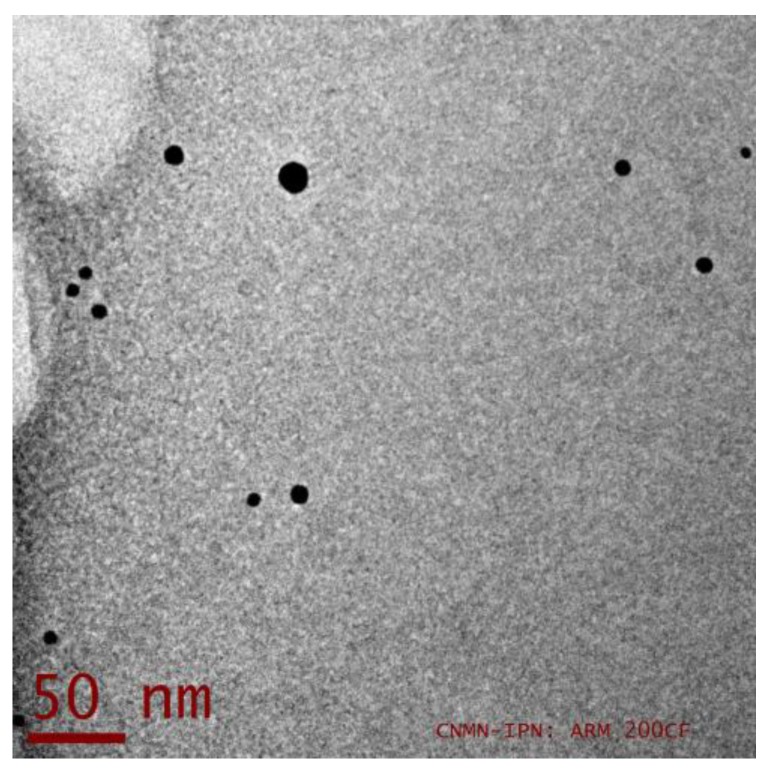
Representative micrograph of the Au-Pt NPs analyzed by TEM.

Figure 3 is a representation of a typical transmission electronic microscopy (TEM) image in bright field mode obtained in the studied sample. The dark points correspond to the isolated bimetallic NPs that can be noticed with a quasi-spherical morphology without agglomerations or cluster formation. 

**Figure 4:**
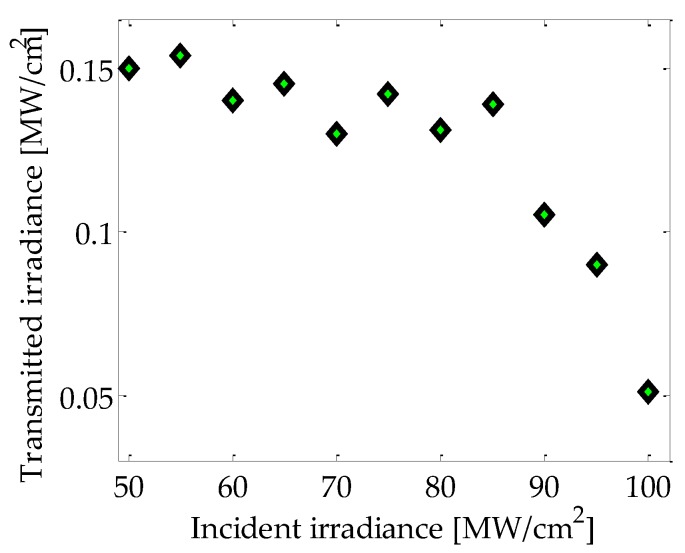
Experimental results for the transmitted irradiance vs. incident irradiance in the sample interacting with nanosecond pulses at 532 nm wavelength.

In Figure 4 experimental data are plotted that point out the possibility to modify the optical transmitted irradiance in the sample by using nanosecond pulses at a wavelength of 532 nm, inducing high irradiance interactions. A strong decrease in the optical transmittance as a function of the incident irradiance in the sample can be clearly observed. It can be considered that this behavior is the signature of a two-photon absorption effect describing a third-order nonlinear optical absorption phenomenon [36].

**Figure 5:**
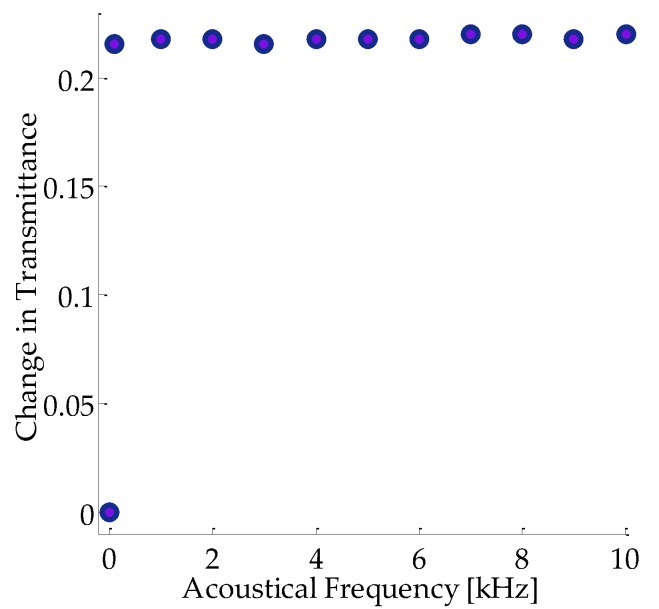
Experimental results describing the change in optical transmittance vs. frequency of acoustical waves in propagation through the sample studied. The optical signals were provided by nanosecond pulses at a wavelength of 532 nm.

Figure 5 shows a remarkable change in the magnitude of the optical transmittance by the influence of acoustical waves in propagation through the sample. In this case the acoustical waves create an important decrease in the transmitted irradiance that can be employed for the identification of mechanical waves. This modification can be regulated by both optical irradiance parameters and the acoustical frequency of interaction. It is worth noting that the sensitivity of the system presents a significant influence by the geometry of the system that may heighten or inhibit acousto-plasmonic resonances.

**Figure 6:**
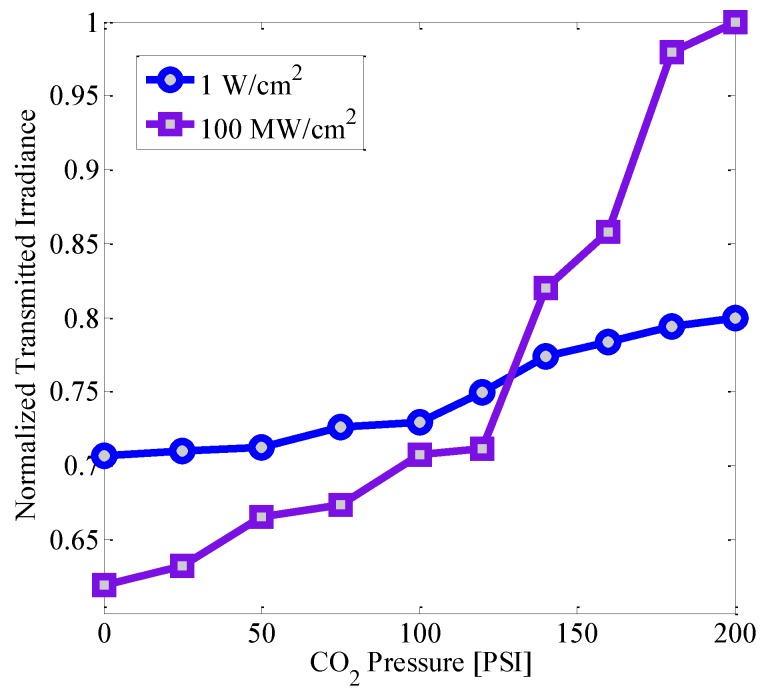
Experimental results illustrating a change in the transmitted irradiance when the sample interacts with CO_2_ at variable pressure and light waves with different intensities.

To further investigate the sensing properties of the Au-Pt NPs, their potential to be employed for the instrumentation of mechanical signals related to the presence of fluids was analyzed. A controlled flux of CO_2_ was incorporated in the experimental setup for monitoring the optical effects as it is illustrated in Figure 1. Outstandingly, the results showed in Figure 6 pointed out a noticeable enhancement of the sensitivity of the system with dependence on interacting irradiance. Regarding the observations described in Figure 4, it is assumed that the results depicted in Figure 6 for high irradiance correspond to a cooperative contribution related to the two-photon absorption effects exhibited by the NPs. 

Single-beam transmittance experiments for CO_2_ detection by Au-Pt NPs were carried out by the fundamental harmonic of our Nd:YAG system (1064 nm wavelength) and the absence of a notable participation of the nanostructures in the results was observed. Acoustical signals interacting with nonlinear optical properties excited near the absorption peak related to one of the SPR bands of the sample can be associated with collective plasmonic excitations [37]. The propagation and absorption of the sample can be affected by molecular behavior and composition of their surroundings [8,38]. Then, comparative experiments were undertaken in order to guarantee that the key role of the absorptive response of acoustical and optical waves can be mainly associated with the Au-Pt NPs together to a small contribution of the ethanol or the dielectric cuvette. The results revealed that nonlinear optical absorption effects of the Au-Pt NPs are at least three orders of magnitude higher than the magnitudes that corresponded to ethanol or quartz. Changes in acoustic resonances related to the geometry of the container were calculated and experimentally evaluated to distinguish the acoustic absorption mechanisms that concern the quartz cuvette and ethanol. In this work the fascinating nonlinear and plasmonic effects in bimetallic NPs that drastically differ from other advanced materials is highlighted. Potential applications for ultrafast and non-contact detection of mechanical signals by all-optical micromachines are attractive. 

## 4. Conclusions

A strong enhancement in gas sensing properties exhibited by Au-Pt NPs were observed when two-photon absorption nonlinearities are involved in optical measurements. Regarding that nonlinear effects in metallic NPs are closely related to plasmonic phenomena, the superposition of both acoustical and nonlinear optical interactions in bimetallic NPs can be proposed for designing nonlinear mechano-optical functions. Bimetallic hybrid nanostructures driven by nonlinear processes seem to be good candidates for designing tunable energy transfer mechanisms. Considering biocompatibility and high sensitivity related to Au-Pt NPs, potential applications for developing plasmonic instrumentation devices suitable for biomedical monitoring can be contemplated.
